# Allostatic Load, Single, and Dual Chronic Conditions: Evidence from the National Health and Nutrition Examination Survey

**DOI:** 10.1089/met.2021.0008

**Published:** 2022-03-15

**Authors:** Peter Memiah, Sibhatu Biadgilign, Jamie Kuhlman, Courtney Cook, Piera Mburia, Carol Kingori, Daniel Sarpong, Gabriel Buluku, Marquis Hawkins

**Affiliations:** ^1^ Division of Epidemiology and Prevention, Institute of Human Virology, University of Maryland School of Medicine, Baltimore, Maryland, USA.; ^2^ Friedman School of Nutrition Science and Policy, Tufts University, Boston, Massachusetts, USA.; ^3^ College of Health Sciences and Professions, Ohio University, Athens, Ohio, USA.; ^4^ Department of Nursing, Fortis Institute, Pensacola, Florida, USA.; ^5^ Department of Public Health, University of Reno, Reno, Arizona, USA.; ^6^ Centre of for Minority Health and Health Disparities Research and Education, Xavier University of Louisiana, New Orleans, Louisiana, USA.; ^7^ Department of Medicine, University of Maryland School of Medicine Midtown Campus, Baltimore, Maryland, USA.; ^8^ Department of Epidemiology, University of Pittsburgh, Pittsburgh, Pennsylvania, USA.

**Keywords:** allostatic load, diabetes, cardiovascular disease, NHANES, race

## Abstract

**Background:**

Allostatic load (AL) is defined as a cumulative burden of chronic stress and life events, which involves the interaction of different physiological systems at varying degrees of activity. AL is suspected of contributing to health disparities among different populations. Suppressed or overactive physiological systems can interrupt AL affecting proper tissue and organ function leading to disease. The objective of our study was to determine the association of AL with dual chronic conditions.

**Methods:**

We used data from the National Health and Nutrition Examination Survey (NHANES). For the current analysis, we used the data cycles of 2007–2010, which is the most recent data that collected comprehensive measures of the composite AL outcome variable. Descriptive, bivariate, and multivariable logistic regression, with stepwise forward variable selection method (*P* < 0.05), were conducted using STATA/IC 15.0.

**Results:**

AL levels were high among 20% of the respondents (*n* = 2179). Having a lower income to poverty ratio, being married, physical inactivity, experiencing sleep problems, and a history of smoking were significantly associated with high AL (*P* < 0.05). Non-Hispanic blacks [odds ratio (OR): 1.8; 95% confidence interval (CI): 1.6–2.4] and Mexicans and other Hispanics (OR: 1.4; 95% CI: 1.1–1.7) had higher AL compared to Caucasians. Having cardiovascular disease (CVD) (OR: 1.7; 95% CI: 1.4–2.2) and diabetes (OR: 4.7; 95% CI: 3.8–5.7) independently, as well as both CVD and diabetes (OR: 3.1; 95% CI 2.7–3.6), were associated with higher odds of AL. We conducted an age-adjusted regression model that indicated higher odds of elevated AL among females with diabetes independently (OR: 1.4; 95% CI: 1.2–1.9) and with both CVD and diabetes (OR: 1.6; 95% CI: 1.2–2.1) compared to men.

**Conclusions:**

Despite the significant impact and association of AL with overall health, there is minimal evidence of its risk factors and linkage to disease burden. Modifiable lifestyle factors were associated with a higher AL. There is a critical need to support ethnic and gender contextual interventions to reduce the burden of AL on chronic conditions.

## Introduction

Allostatic load (AL) refers to the cumulative burden of chronic stress and life events. It involves the interaction of different physiological systems at varying degrees of activity. When environmental challenges exceed the individual ability to cope, then allostatic overload ensues. The health disparities with risk factors lead to allostatic dysregulation with maladaptive physiological (including hypothalamo-pituitary-adrenal axis, sympathetic nervous system, and the immune system) and behavioral responses culminating in allostatic loading and, ultimately, to diseased states such as with cardiovascular disease (CVD).^1^ AL is identified by the use of biomarkers and clinical criteria.

Chronic disease leads to poor overall health, disability, and death, accounting for most health care expenditures in the United States.^2,3^ Approximately half (50.9%) of adults have at least one chronic condition, with 26% having two or more chronic conditions.^4^ Cross sectional evidence shows that high AL is associated with CVD,^5,6^ periodontal disease,^6^ chronic fatigue syndrome, and diabetes.^7^ In longitudinal studies, high AL is predictive of all-cause mortality^8^ and lower cognitive and physical functioning.^9^ With limited evidence on whether AL is higher among those with multiple chronic diseases or not, this study aims to examine the association of AL with CVD or diabetes or both conditions using the National Health and Nutrition Examination Survey (NHANES) study. We further assess race/ethnic comparisons while adjusting for potential confounders.

## Methods

### Study design and population

NHANES is a cross sectional, observational study of noninstitutionalized U.S. residents conducted by the National Center for Health Statistics of the Centers for Disease Control and Prevention (CDC). NHANES uses a stratified, multistage probability design to obtain a nationally representative sample of the U.S. population. Non-Hispanic black and Hispanic individuals, persons 60 years of age and older, and individuals of low-income are oversampled to produce those nationally representative estimates.^7^ We used the most recent NHANES data cycles of 2007–2010, with complete measures of AL, described in detail below. We restricted our sample to participants with data on the primary outcome variables (*i.e.*, diabetes and CVD). The National Center for Health Statistics Institutional Review Board approved this protocol. All participants provided written informed consent at the time of the household interview.

### Allostatic load

All measures for AL were collected using a mobile examination center by a trained technician using standardized procedures. AL was calculated as the sum of 11 indicators of biological functioning across a range of regulatory systems, including systolic blood pressure (SBP) (≥140 mmHg) and diastolic blood pressure (>90 mmHg) (cardiovascular system); plasma high-density lipoprotein cholesterol (HDL-C) (<30 mg/dL), triglycerides (≥150 mg/dL), total cholesterol (TC) (≥250 mg/dL), serum albumin (≤3.9 mg/dL), c-reactive protein (≥0.33 mg/dL), fibrinogen (>336 mg/dL), creatinine clearance (≤16.49 mg/dL), and glycohemoglobin (>5.6%) (lipid metabolism and long-term atherosclerotic risk); and body mass index (BMI) (≥30 kg/m^2^) (adipose tissue deposition).^10^ High AL was defined as having three or more of these indicators.

### Diabetes

Diabetes was defined as self-reported physician-diagnosed diabetes. Participants were asked to report the age they were diagnosed. Duration of diabetes was categorized as 5 or younger, 5 to 9, and 10 or more years.

### Cardiovascular disease

CVD was defined as a self-reported history of congestive heart failure, coronary heart disease, angina, myocardial infarction, or stroke.

### Covariates

Demographic characteristics were collected through self-report during the household examination and included age (20–29, 30–49, 50–64, 65–74, ≥75 years), race/ethnicity (non-Hispanic white, non-Hispanic black, Hispanic (*i.e.*, Mexican American and other Hispanic), and other – including biracial), marital status (married/living with partner, single), and income to poverty ratio (<1.3, 1.3–3.5, >3.5). The income to poverty ratio was created using the Department of Health and Human Services poverty guidelines.^11^ The participants' family monthly income was divided by the poverty guidelines, which takes into account family size.

Behavioral characteristics included physical activity (PA) (inactive, insufficiently active, meeting PA (active and highly active) guidelines),^12^ self-reported trouble sleeping, current smoking status (nonsmoker, former smoker, never smoker), and alcohol intake (nondrinker, moderate drinker, excessive drinker). PA was assessed using a modified Global Physical Activity Questionnaire.^8^ Participants were asked to report the frequency and duration of engaging in any vigorous-intensity sports, fitness, or recreational activities that cause large increases in breathing or heart rate like running or basketball for at least 10 min continuously in a typical week. We defined participants as inactive if they reported no PA, insufficiently active if they reported >0 to <150 min/week of PA, and meeting guidelines for PA if they reported ≥150 min/week of PA.^12^

Alcohol intake was assessed as the average number of alcohol drinks/day in the past 12 months. We defined participants as nondrinkers, if they reported not drinking in the past 12 months or in their lives, and moderate drinkers, if they reported drinking less than 4 or 5 drinks/day in men and women. Participants were defined as excessive drinkers if they drank more than 4 or 5 drinks/day in men and in women.^13^

Other health conditions included self-reported doctor-diagnosed liver disease, cancer, weak/failing kidneys, and general health status. General health condition was defined as self-reported excellent/very good, good/fair, or poor health. Participants also reported the number of days they did not have good physical or mental health in the past month.

### Statistical analysis

We used descriptive statistics to compare characteristics of the population by diabetes and by CVD status. Chi-square was used for categorical variables and Student's *t*-test for continuous variables. For the main analysis, multivariable logistic regression, with stepwise forward variable selection method (*P* < 0.05) was used to compare the odds of high AL by race and age stratified by gender. We repeated these analyses with diabetes and CVD status as the independent variables, adjusting for age. To test for effect modification, we stratified the analyses by gender and race/ethnicity. The complex survey design used for NHANES data collection was incorporated into all data analysis using STATA/IC 15.0 (StataCorp LP: College Station, TX). All analyses were conducted in 2018 and considered statistically significant at an alpha of 0.05.

## Results

Table 1 describes the demographic characteristics of the population by chronic disease status. Age, ethnicity/race, and income to poverty ratio were significantly (*P* < 0.001) associated with having CVD, diabetes, and both chronic conditions (CVD and diabetes). Specifically, a significantly (*P* < 0.001) higher proportion of respondents 50–64 years old had CVD, diabetes, and both chronic conditions (30.5%, 37%, and 34%, respectively) versus those 20–29 years old (1.7%, 2.6% and 2.5%, respectively). There were significant (*P* < 0.001) gender differences in respondents who reported having CVD with 56.2% being men and 43.9% women, but no significant gender differences were observed in respondents who had CVD, diabetes, and both chronic conditions (Table 1).

**Table 1.** Association of Cardiovascular Disease, Diabetes, and Both Chronic Conditions Across Demographic Characteristics

Variable	CVD			Diabetes			CVD/diabetes		
	No* n *(%)	Yes* n *(%)	(*P*-value)	No* n *(%)	Yes* n *(%)	(*P*-value)	No* n *(%)	Yes* n *(%)	(*P*-value)
Age, years									
20–29	1652 (20.4)	15 (1.7)	(<0.001^**^)	1650 (20.4)	17 (2.6)	(<0.001^**^)	1635 (21.6)	32 (2.5)	2590.38 (<0.001^**^)
30–49	3422 (40.8)	113 (13.3)		3351 (40.2)	184 (20.4)		3262 (41.9)	273 (18.1)	
50–64	2346 (25.6)	306 (30.5)		2198 (25)	454 (37.3)		2002 (24.6)	650 (34.6)	
65–74	1069 (8.3)	313 (26)		1036 (8.4)	346 (25)		837 (73.4)	545 (24.4)	
>75	701 (4.9)	357 (28.5)		851 (6.1)	207 (14.8)		595 (4.6)	463 (20.4)	
Gender									
Male	4431 (47.7)	666 (56.2)	(<0.001^**^)	4483 (48.3)	614 (49.7)	(0.542)	4011 (47.7)	1086 (52.9)	27.540 (0.008)
Female	4842 (52.3)	472 (43.9)		4699 (51.7)	615 (50.3)		4388 (52.3)	926 (47.1)	
Race									
Non-Hispanic white	4379 (69.3)	693 (77.2)	(<0.001^**^)	4585 (70.5)	487 (63.1)	(<0.001^**^)	4072 (70)	1000 (70)	43.560 (0.011^**^)
Non-Hispanic black	1641 (10.3)	204 (10.8)		1545 (9.8)	300 (15.7)		1423 (9.8)	422 (13)	
Mexican and Other Hispanic	2808 (14.1)	201 (7.3)		2622 (13.5)	387 (14)		2501 (14)	508 (11)	
Other	445 (6.4)	40 (4.8)		430 (6.2)	55 (7.2)		403 (6.2)	82 (6)	
Income to poverty ratio									
<1.3	2633 (20.1)	370 (26.1)	(<0.001^**^)	2618 (20.3)	385 (24.1)	(<0.001^**^)	2376 (20)	627 (24.4)	115.525 (<0.001^**^)
1.3–3.5	3202 (35.4)	433 (41.5)		3170 (35.3)	465 (42.6)		2870 (35)	765 (41.5)	
>3.5	2620 (44.5)	233 (32.4)		2600 (44.4)	253 (33.3)		2426 (45)	427 (34.1)	
Marital status									
Single	3613 (35.3)	490 (38.5)	(0.084)	3592 (35.3)	511 (37.7)	(0.196)	3270 (35.3)	833 (36.9)	2.799 (0.276)
Married/living with partner	5655 (64.7)	648 (61.4)		5587 (64.7)	716 (62.3)		5126 (64.7)	1177 (63.1)	

^**^ *P* < 0.001.

CVD, cardiovascular disease.

### Association of CVD, diabetes, and both chronic conditions across behavioral characteristics

Table 2 describes the behavioral characteristics of the population by chronic disease status. Participants who met guidelines for PA had significantly (*P* < 0.001) lower prevalence of CVD (7%), diabetes (6.3%), and both chronic conditions (7.2%) than physically inactive participants (63.2%, 66.1%, and 63%, respectively). Among the respondents who reported sleep problems, a significantly (*P* < 0.001) higher proportion had CVD, diabetes, and both chronic conditions (41.5%, 34.8%, and 36.6%, respectively) compared to those who did not (58.5%, 65.2%, and 63.4%, respectively) (Table 2).

**Table 2.** Association of Cardiovascular Disease, Diabetes, and Both Chronic Conditions Across Behavioral Characteristics

Variable	CVD			Diabetes			CVD/diabetes		
	No* n *(%)	Yes* n *(%)	(*P*-value)	No* n *(%)	Yes* n *(%)	Test statistic (*P*-value)	No* n *(%)	Yes* n *(%)	Test statistic (*P*-value)
PA									
Inactive	4958 (46.1)	779 (63.2)	(<0.001^**^)	4858 (45.8)	879 (66.1)	(<0.001^**^)	4357 (45)	1380 (63)	(<0.001^**^)
Insufficiently active	3016 (37.3)	299 (29.8)		3017 (37.5)	298 (27.6)		2788 (37.8)	527 (29.8)	
Meeting guidelines	1288 (16.6)	59 (7)		1295 (16.7)	52 (6.3)		1243 (17.2)	104 (7.2)	
Sleep problems									
No	7207 (76.2)	671 (58.5)	(<0.001^**^)	7067 (75.7)	811 (65.2)	(<0.001^**^)	6585 (76.7)	1293 (63.4)	(<0.001^**^)
Yes	2062 (23.8)	466 (41.5)		2110 (24.4)	418 (34.8)		1810 (23.3)	718 (36.6)	
Smoking									
Nonsmoker	5082 (55.2)	435 (39.5)	(<0.001^**^)	4906 (54.4)	611 (48.7)	(<0.001^**^)	4610 (55.4)	907 (44.7)	(<0.001^**^)
Past	2143 (23.3)	463 (40.4)		2185 (23.8)	421 (34.7)		1874 (22.7)	732 (37.1)	
Current	2043 (21.5)	240 (20.2)		2087 (21.8)	196 (16.6)		1911 (21.9)	372 (18.2)	
Alcohol									
Nondrinker	2700 (25.7)	522 (45)	308.635 (<0.001^**^)	2585 (25.2)	637 (50.6)	(<0.001^**^)	2260 (24)	962 (46)	(<0.001)
Moderate drinking	4711 (60.1)	466 (48.8)		4738 (60.6)	439 (42.9)		4389 (61)	788 (47)	
Excessive drinking	1280 (14.2)	73 (6.2)		1269 (14.2)	84 (6.5)		1212 (15)	141 (7)	

^**^ *P* < 0.001.

PA, physical activity.

### Association of CVD, diabetes, and both chronic conditions across health-related conditions and biomarkers

Table 3 indicates that respondents who reported having liver disease, cancer, and weak or failing kidneys had a significant association with CVD, diabetes, and both CVD and diabetes (*P* < 0.001). Other factors that were significantly associated with the three (CVD, diabetes, and both chronic diseases) included respondent perception of physical health and general health; obesity as measured by BMI; low-density lipoprotein (LDL) cholesterol; HDL-C; triglycerides; SBP; hypertension; albumin; CRP; and AL (*P* < 0.001).

**Table 3.** Association of Cardiovascular Disease, Diabetes, and Both Chronic Conditions Across Health-Related Conditions and Biomarkers

Variable	CVD			Diabetes			CVD/diabetes		
	No* n *(%)	Yes* n *(%)	(*P*-value)	No* n *(%)	Yes* n *(%)	(*P*-value)	No* n *(%)	Yes* n *(%)	(*P*-value)
Liver diseases									
No	8984 (97.5)	1058 (93.5)	87.014 (<0.001^**^)	8881 (97.3)	1161 (95.2)	25.067 (0.012^**^)	8150 (97.6)	1892 (94.4)	88.683 (<0.001^**^)
Yes	277 (2.6)	75 (6.5)		290 (2.7)	62 (4.8)		240 (2.4)	112 (5.6)	
Cancer									
No	8513 (91.9)	878 (76.9)	409.999 (<0.001^**^)	8349 (91.3)	1042 (83.8)	102.112 (<0.001^**^)	7743 (92.2)	1648 (81.2)	359.228 (<0.001^**^)
Yes	751 (8.1)	258 (23.1)		825 (8.7)	184 (16.2)		650 (7.8)	359 (18.8)	
Weak/failing kidneys									
No	9095 (98.7)	1035 (91.9)	406.109 (<0.001^**^)	9001 (98.6)	1129 (93.6)	214.022 (<0.001^**^)	8269 (98.9)	1861 (93.8)	361.916 (<0.001^**^)
Yes	163 (1.3)	101 (8.1)		171 (1.4)	93 (6.4)		120 (1.1)	144 (6.2)	
Physical health not good (past month), days									
<15	7809 (85.8)	820 (74.1)	165.177 (<0.001^**^)	7692 (85.6)	937 (76.9)	90.926 (<0.001^**^)	7108 (86.1)	1521 (77.2)	154.782 (<0.001^**^)
15–30	1464 (14.2)	318 (25.9)		1490 (14.4)	292 (23.1)		1291 (13.9)	491 (22.8)	
Mental health not good (past month), days									
<15	7724 (83.9)	901 (79.3)	24.315 (0.006)	7631 (83.8)	994 (81.1)	8.229 (0.056)	7005 (84.1)	1620 (80.5)	22.869 (0.003^**^)
15–30	1549 (16.1)	237 (20.7)		1551 (16.2)	235 (18.9)		1394 (15.9)	392 (19.5)	
General health condition									
Excellent, very good	3338 (46.9)	190 (20.2)	748.295 (<0.001^**^)	3360 (47.2)	168 (17.1)	863.912 (<0.001^**^)	3199 (48.8)	329 (20.2)	1219.544 (<0.001^**^)
Good/fair	5112 (50.9)	745 (68.7)		4995 (50.7)	862 (71.8)		4495 (49.5)	1362 (69.9)	
Poor	261 (2.2)	136 (11.1)		261 (2.1)	136 (11.1)		184 (1.7)	213 (9.9)	
Duration of diabetes, years									
<5	8688 (95.9)	877 (80.3)	748.997 (<0.001^**^)	9182 (100)	383 (34.9)	1300.4 (<0.001^**^)	8399 (100)	1166 (62.1)	7120.757 (<0.001^**^)
5 to 9	201 (1.4)	83 (6.1)		0 (0)	284 (21.4)		0 (0)	284 (12.5)	
≥10	384 (2.7)	178 (13.6)		0 (0)	562 (43.7)		0 (0)	562 (25.5)	
Obesity									
No, BMI <30 kg/m^2^	5914 (66.3)	618 (54.7)	92.404 (<0.001^**^)	6038 (67.9)	494 (36.8)	672.445 (<0.001^**^)	5556 (68.3)	976 (47.6)	477.117 (<0.001^**^)
Yes, BMI ≥30 kg/m^2^	3359 (33.7)	520 (45.3)		3144 (32.1)	735 (63.2)		2843 (31.7)	1036 (52.4)	
Waist to height ratio									
03 to <0.5	1610 (21.2)	67 (6.7)	347.237 (<0.001^**^)	1649 (21.5)	28 (3.8)	837.738 (<0.001^**^)	1587 (22.3)	90 (5.8)	867.949 (<0.001^**^)
0.5 to <0.7	6374 (68.7)	772 (71.3)		6348 (69.2)	798 (6.6)		5794 (68.8)	1352 (69.6)	
≥0.7	1099 (10.1)	238 (22)		991 (9.4)	346 (30.4)		859 (8.9)	478 (24.6)	
HbA1C (%)									
<6.5	8428 (93.8)	838 (76.9)	649.435 (<0.001^**^)	8830 (97.5)	436 (37.1)	8413.413 (<0.001^**^)	8110 (97.8)	1156 (60.3)	5127.414 (<0.001^**^)
6.5 to <7.5	453 (3.5)	177 (14.1)		248 (1.9)	382 (31.6)		200 (1.6)	430 (20.7)	
7.5 to 8.9	206 (1.6)	81 (5.8)		52 (0.4)	235 (18.9)		42 (0.3)	245 (11.5)	
≥9	186 (1.1)	42 (3.2)		52 (0.3)	176 (12.4)		47 (0.3)	181 (7.5)	
Total cholesterol (mg/dL)									
Normal, <200	7932 (85.7)	1003 (88.4)	9.693 (0.075)	7835 (85.6)	1100 (89.8)	23.000 (0.005)	7155 (85.4)	1780 (88.7)	22.949 (0.018^**^)
High, ≥200	1341 (14.3)	135 (11.6)		1347 (14.5)	129 (10.2)		1244 (14.6)	232 (11.3)	
LDL cholesterol (mg/dL)									
Normal, <100	7120 (76.7)	785 (68.9)	51.949 (<0.001^**^)	7052 (76.8)	853 (67.7)	69.907 (<0.001^**^)	6493 (77.2)	1412 (69.3)	84.858 (<0.001^**^)
High, ≥100	2153 (23.3)	353 (31.1)		2130 (23.2)	376 (32.2)		1906 (22.8)	600 (30.7)	
HDL-C (mg/dL)									
Low, <40	1412 (31.9)	307 (55.7)	297.646 (<0.001^**^)	1409 (31.8)	310 (57)	331.136 (<0.001^**^)	1215 (30.5)	504 (54.5)	482.174 (<0.001^**^)
Normal, ≥40	2979 (68.1)	248 (44.3)		2960 (68.2)	267 (43)		2775 (69.5)	452 (45.5)	
Triglycerides (mg/dL)									
Normal, <150	3202 (72.8)	352 (62.4)	63.891 (<0.001^**^)	3202 (73.4)	352 (57.2)	157.039 (<0.001^**^)	2953 (73.9)	601 (60.7)	163.009 (<0.001^**^)
High, ≥150	1278 (27.2)	213 (37.6)		1240 (26.6)	251 (42.8)		1107 (26.1)	384 (39.3)	
Systolic blood pressure (mmHg)									
Normal, <140	7839 (88.1)	803 (73.9)	274.545 (<0.001^**^)	7780 (88.2)	862 (73.6)	292.424 (<0.001^**^)	7207 (88.9)	1435 (74.9)	434.219 (<0.001^**^)
High, ≥140	1434 (11.9)	335 (26.1)		1402 (11.8)	367 (26.4)		1192 (11.1)	577 (25.1)	
Diastolic blood pressure (mmHg)									
Normal, <90	8820 (95.6)	1075 (94.4)	4.635 (0.252)	8729 (95.5)	1166 (94.7)	2.729 (0.255)	7992 (95.6)	1903 (94.6)	6.492 (0.155)
High, ≥90	453 (4.4)	63 (5.6)		453 (4.5)	63 (5.3)		407 (4.4)	109 (5.4)	
Heart rate (beats/minute)									
Normal, <90	8333 (90)	1032 (92)	6.734 (0.102)	8310 (90.6)	1055 (86)	37.262 (0.002^**^)	7588 (90.4)	1777 (89)	5.769 (0.192)
High, ≥90	940 (10)	106 (8)		872 (9.4)	174 (14)		811 (9.6)	235 (11)	
Hypertension									
No	6270 (72.7)	305 (31)	1266.884 (<0.001^**^)	6239 (72.8)	336 (30.2)	1334.5670 (<0.001^**^)	5997 (75.3)	578 (32.6)	2154.929 (<0.001^**^)
Yes	3003 (27.3)	833 (69)		2943 (27.2)	893 (69.8)		2402 (24.7)	1434 (67.4)	
Albumin (g/dL)									
Low, <3.8	8461 (92.9)	970 (86.4)	95.782 (<0.001^**^)	8409 (93.1)	1022 (84.6)	161.235 (<0.001^**^)	7725 (93.4)	1706 (86.2)	189.581 (<0.001^**^)
Normal ≥3.8	812 (7.1)	168 (13.6)		773 (76.9	207 (15.4)		674 (6.6)	306 (13.8)	
CRP (mg/dL)									
Normal, <0.3	5933 (67.7)	616 (55.6)	103.052 (<0.001^**^)	5902 (68)	647 (52.5)	168.859 (<0.001^**^)	5456 (68.7)	1093 (54.9)	216.738 (<0.001^**^)
High, ≥0.3	3340 (32.3)	522 (44.4)		3280 (32)	582 (47.5)		2943 (31.3)	919 (45.1)	
Allostatic load									
Normal, <3	7094 (80)	701 (64.8)	214.968 (<0.001^**^)	7254 (81.8)	541 (45.8)	1215.600 (<0.001^**^)	6693 (82.3)	1102 (57.4)	933.722 (<0.001^**^)
High, ≥3	2179 (20)	437 (35.2)		1928 (18.2)	688 (54.2)		1706 (17.7)	910 (42.6)	

^**^ *P* < 0.001.

BMI, body mass index; CRP, C-reactive protein; HbA1C, hemoglobin A1c; HDL, high-density lipoprotein cholesterol; LDL, low-density lipoproteins.

### Correlates of CVD, diabetes, or both chronic diseases

As shown in Table 4, non-Hispanic blacks were almost two times more likely to have a higher AL compared to the non-Hispanic white population (adjusted odds ratio [aOR] = 1.99, 95% confidence interval [CI]: 1.64–2.41), while Mexicans and other Hispanics were 1.42 times more likely to have both a higher AL compared to the non-Hispanic white population (aOR = 1.42, 95% CI: 1.13–1.77). Having an income to poverty ratio of <1.3 was (24%) less likely associated with high AL (aOR = 0.76, 95% CI: 0.65–0.89) compared to having an income to poverty ratio of greater than 3.5. With regard to marital status, the odds of having a lower AL were significantly (18%) lower among respondents whose marital status was single (aOR = 0.82, 95% CI: 0.75–0.91) compared to those who were married (Table 4).

**Table 4.** Predictive Factors Associated with Allostatic Load

Variables	Unadjusted			Adjusted		
	OR	95% CI	*P*	OR	95% CI	*P*
Race						
Non-Hispanic white	1.00			1.00		
Non-Hispanic black	1.81	1.48–2.21	<0.001^**^	1.99	1.64–2.41	<0.001^**^
Mexican and Other Hispanic	1.23	1.00–1.53	0.050	1.42	1.13–1.77	0.003^**^
Other	0.67	0.47–0.96	0.030	0.74	0.51–1.07	0.102
Income to poverty ratio						
<1.3	1.82	1.70–1.96	0.016^**^	1.76	1.65–1.89	0.001^**^
1.3 to 3.5	1.58	1.49–1.68	<0.001^**^	1.54	1.46–1.64	<0.001^**^
>3.5	1.00			1.00		
Marital status						
Single	0.84	0.77–0.92	0.001^**^	0.82	0.75–0.91	<0.001^**^
Married/Living w/Partner	1.00			1.00		
Behavioral						
PA						
Inactive	1.51	1.47–1.57	<0.001^**^	1.54	1.49–1.60	<0.001^**^
Insufficiently active	1.28	1.21–1.37	<0.001^**^	1.32	1.24–1.42	<0.001^**^
Meeting guidelines	1.00			1.00		
Sleep problems						
Yes	1.44	1.26–1.66	<0.001^**^	1.39	1.21–1.59	<0.001^**^
No	1.00			1.00		
Smoking						
Current	1.28	1.08–1.52	0.005	1.13	0.96–1.34	0.139
Past	1.30	1.13–1.49	0.001^**^	1.40	1.21–1.62	<0.001^**^
Nonsmoker	1.00			1.00		
Alcohol						
Nondrinker	0.50	0.44–0.57	<0.001^**^	0.54	0.47–0.62	<0.001^**^
Moderate drinking	0.68	0.59–0.77	<0.001^**^	0.85	0.73–0.98	0.024
Excessive drinking	1.00			1.00		
Weak/failing kidneys						
Yes	1.00			1.00		
No	0.42	0.32–0.55	<0.001^**^	0.47	0.37–0.62	<0.001^**^
Physical health not good (past month), days						
<15	1.73	1.53–1.95	<0.001^**^	1.67	1.38–1.89	<0.001^**^
15 to 30	1.00			1.00		
Mental health not good (past month), days						
<15	1.32	1.13–1.54	0.001^**^	1.37	1.17–1.60	<0.001^**^
15 to 30	1.00			1.00		
General health condition						
Excellent, very good	1.00			1.00		
Good/fair	2.62	2.27–3.03	<0.001^**^	2.58	2.24–2.97	<0.001^**^
Poor	6.69	4.91–9.12	<0.001^**^	6.30	4.62–8.60	<0.001^**^
Duration of diabetes, years						
<5	4.81	3.17–7.29	<0.001^**^	4.11	2.71–6.25	<0.001^**^
5 to 9	5.02	3.93–6.40	<0.001^**^	4.28	3.37–5.43	<0.001^**^
≥10	1.00			1.00		
Waist to height ratio						
03 to <0.5	1.00			1.00		
0.5 to <0.7	11.88	7.59–18.60	<0.001^**^	11.16	7.07–17.62	<0.001^**^
≥0.7	85.18	53.40–135.88	<0.001^**^	79.96	49.44–129.32	<0.001^**^
HDL-C, mg/dL						
Low, <40	1.40	1.17–1.66	<0.001^**^	1.40	1.18–1.66	<0.001^**^
Normal, ≥40	1.00			1.00		
Triglycerides (mg/dL)						
Normal, <150	3.63	3.06–4.32	<0.001^**^	3.57	2.99–4.26	<0.001^**^
High, ≥150	1.00			1.00		
Chronic conditions						
CVD						
Yes	2.16	1.77–2.65	<0.001^**^	1.74	1.39–2.18	<0.001^**^
No	1.00			1.00		
Diabetes						
Yes	5.17	4.18–6.41	<0.001^**^	4.67	3.79–5.74	<0.001^**^
No	1.00			1.00		
CVD/diabetes						
Yes	3.40	2.94–3.94	<0.001^**^	3.13	2.71–3.62	<0.001^**^
No	1.00			1.00		

^**^ *P* < 0.001. Adjusted for age, gender, and other health conditions (liver diseases and cancer).

CI, confidence interval; OR, odds ratio.

Respondents who reported being physically inactive were more likely to have higher AL (aOR = 1.54, 95% CI: 1.49–1.60) compared to those who reported meeting the physical exercise guidelines. Respondents who reported experiencing sleep problems were significantly (aOR = 1.39, 95% CI: 1.21–1.59) more likely to have a higher AL compared to those who reported having no sleep problems. Respondents who reported being past smokers were significantly more likely (aOR = 1.40, 95% CI: 1.21–1.62) to have a higher AL compared to the nonsmokers. Nondrinkers were less likely (aOR = 0.54, 95% CI: 0.46–0.62) to have a higher AL compared to excessive drinkers. Respondents with no weak/failing kidneys were (53%) less likely (aOR = 0.47, 95% CI: 0.37–0.42) to have a higher AL compared to those with weak/failing kidneys.

Those who reported not having good mental health and physical health in the past 2 weeks were more likely to have a higher AL (aOR = 1.67, 95% CI: 1.38–1.89 and aOR = 1.37, 95% CI: 1.17–1.60, respectively). Low HDL-C was associated with higher AL (aOR = 1.40, 95% CI: 1.18–1.66). Those who had CVD were more likely to have a higher AL (aOR = 1.74, 95% CI: 1.39–2.18); furthermore, respondents who had diabetes were more likely to have a higher AL (aOR = 4.67, 95% CI: 3.79–5.74).

Finally, those with both conditions (CVD and diabetes) were more likely to have a higher AL compared to those who had neither (aOR = 3.13, 95% CI: 2.71–3.62) (Table 4). We further conducted an age-adjusted analysis model whereby there were higher odds of elevated AL among females with diabetes independently (OR: 1.4; 95% CI: 1.2–1.9) and women with both CVD and diabetes (OR: 1.6; 95% CI: 1.2–2.1) compared to men.

## Discussion

The findings revealed that there is a significant association of AL with diabetes, CVD, and having both conditions. The association between high AL and CVD in our study is also evident in other similar settings that show increased AL (excluding respective definitive parameters) being significantly associated with higher odds of hypertension, diabetes, and self-reported CVD.^5^ The presence of AL mainly manifests a greater array of varied health outcomes such as CVD and mortality.^14^

The study findings indicate that being a past smoker and having excessive drinking habits increase the chance of having high AL. Recent literature associates frequent alcohol use with components of AL.^15^ Reciprocally, the higher level of AL compromises positive health behaviors through stress experiences and through damaging behaviors such as tobacco and alcohol abuse that frequently accompany chronic stress states.^16^ Similarly, there is a link between physiological indicators of stress to future morbidity and mortality from cardiometabolic disorders such as diabetes.^17^ Redundant exposure to stress demonstrates excess secretion of glucocorticoids and catecholamines and increases the risk for diabetes and CVD.^18^ This reflects on AL score with a direct measure of stress-induced cardiovascular, metabolic, and immune biomarkers resulting in greater physiological dysfunction.^18,19^

In our study, lower income or poverty level was associated with high AL. Other findings have documented that higher socioeconomic status promotes some healthy behaviors and is associated with lower AL.^20^ This finding is also supported by other research findings, in which households with incomes below the poverty line are positively associated with higher AL; this, therefore, presents long-term health implications of living in neighborhoods with high concentrations of poverty apart from the household income level.^21,22^ However, the relationship between poverty and AL may also be mediated through stressors associated with neighborhood conditions but not merely on the psychological stress manifestation.^23^

In this study, those who are physically inactive or do not meet the physical exercise guidelines were reported to have a high AL. It has been documented that individual lifestyle habits such as diet, exercise, substance abuse, and developmental experiences set life-long behavior patterns and physiological reactivity 24 associated with AL. Having low levels of PA and stressful events of daily life elevates and sustains activities of physiological systems that can cause sleep deprivation, overeating, and other health-damaging behaviors, producing the feeling of being “stressed out.”^25^ This finding is also reinforced through current literature indicating that highly active participants had lower AL and inflammatory risk than sedentary participants with meeting versus not meeting physical exercise guidelines.^26^

Our findings indicate that sleep deprivation/having sleeping problems is associated with higher levels of AL. Sleep deprivation and circadian disruption can be stressors, enhancers of other stressors that have consequences for the brain and many body systems, contributing to the cumulative wear and tear on body systems caused by too much stress.^27^ Evidence from the 2005 to 2008 NHANES revealed that after adjustment for sociodemographic and lifestyle factors and depression status, high AL was significantly associated with sleep apnea, snoring, snorting/stopping breathing, prolonged sleep latency, short sleep duration (<6 hr), and diagnosed sleep disorder.^28^

Other findings have demonstrated that inadequate or problematic sleep can be taken as a neurobiological and physiologic stressor.^29^ Others argue that sleep by itself is considered a component to construct AL.^30^ The literature review suggests that in any case, there might be a bidirectional association between AL and sleep disturbances in that sleep deprivation and poor sleep quality associated with stresses may contribute to AL. High AL might also contribute to sleep disturbances.^9^

Having poor mental and physical health was associated with a higher level of AL. This is also evidenced by a study that showed people with high AL rated their physical health much worse and reported a greater overall smoking history and consumption of alcohol.^31^ A national sample of middle-aged and elderly Taiwanese depicted a significant association between biomarkers of stressful experiences and profiles of physical and mental functioning.^32^ Other studies indicate that mental health might also arise as a result of stress experienced from inequalities in social and economic opportunities and environmental conditions,^33^ further explaining the due effect of chronic stress on the mental health functioning of individuals.

### Limitations

The study was a cross sectional survey that does not follow participants over time; hence, it lacks a temporal order of the factors or evaluates causality and does not allow making causal inferences. Due to the nature of the NHANES data, we did not ascertain the willingness of the individuals to participate or who refused to participate, in which there is a possibility that our results could be under- or overestimated reporting. Some of the variables are self-reported and may possess reporting bias unlike the measurement of the biomarkers and physiologic measures of chronic stress. In addition, all biomarkers' measurements were performed only once, so some phenotype and laboratory markers such as the blood pressure and fibrinogen could be biased, as they can easily be modified by temporal diseases or clinical facts. The study also fails to assess the full impact of chronic stress related to health and wellbeing. Finally, the different AL definitions across different studies makes difficult on the comparison of the results across the studies.

## Conclusion

Our study strongly shows the interplay between higher AL and other variables, such as having CVD and diabetes as well as having both CVD and diabetes, being physically inactive or not meeting the physical exercise guidelines, having sleeping problems, being a past or current smoker, excessive drinking habits, weak or failing kidneys, and poor mental health and physical health. It is recommended to focus and act on those modifiable lifestyle behaviors, such as reducing substance use, participating in regular PA, and experiencing psychosocial support interventions to reduce higher AL for averting CVD and diabetes conditions. There is a need to conduct more longitudinal/cohort studies to better measure the manifestation and biomarkers of AL to reduce the burden on chronic conditions.

## Ethics Approval and Consent to Participate

The study used secondary data from Center for Disease Control: The National Health and Nutrition Examination Survey (NHANES) and does not require ethical approval.

## Availability of Data and Materials

NHANES data and reports (https://wwwn.cdc.gov/nchs/nhanes/Default.aspx) are available upon request from the corresponding author.
